# M184V: The Major Determinant of Cooperative ISL Resistance When in Combination with Multiple TAM2 Mutations in HIV-1 Subtype C

**DOI:** 10.3390/v18091046

**Published:** 2026-09-20

**Authors:** Kyla Nel, Maria Antonia Papathanasopoulos, Adriaan Erasmus Basson

**Affiliations:** HIV Pathogenesis Research Unit, Department of Molecular Medicine and Haematology, School of Pathology, Faculty of Health Sciences, University of the Witwatersrand, Parktown, Johannesburg 2193, South Africa; kyla.nel09@gmail.com (K.N.); maria.papathanasopoulos@wits.ac.za (M.A.P.)

**Keywords:** HIV-1, subtype C, reverse transcriptase, antiretroviral therapy (ART), antiretroviral drugs (ARV), islatravir (ISL), NRTI, drug resistance, phenotypic, thymidine analogue mutation (TAM), M184V

## Abstract

Islatravir is a novel nucleoside reverse transcriptase translocation inhibitor recently approved for the treatment of HIV-1 infection in virologically suppressed PLWH. While we previously documented high-level phenotypic resistance to islatravir (ISL) in HIV-1 subtype C variants harboring combinations of type 2 thymidine analogue mutations (TAM2) along with the M184V resistance-associated mutation, the primary contributor to the observed phenotype remains unclear. This report dissects the relative contribution of M184V to ISL resistance in the context of TAM2-containing variants through systematic reversion analysis. We show that reverting mutant 184V to wildtype M184 resulted in substantial sensitization to ISL, with fold-change reductions ranging from 8.1 to 13.4-fold across three TAM2 genetic backgrounds. Contrary to the well-documented antagonism between M184V and TAMs in zidovudine resistance, these two resistance pathways exhibit cooperative rather than antagonistic interactions in the context of ISL resistance. These findings provide critical evidence for refinement of genotypic resistance interpretation algorithms for ISL and have direct implications for the future clinical management of ISL-based antiretroviral therapy in people living with HIV. Importantly, elucidating the mechanistic basis of M184V-TAM2 cooperation would establish a more predictive framework for understanding how complex resistance mutation patterns impact ISL susceptibility and guide treatment decisions in virologically experienced individuals. Considering the diminished use of TAM2-seletive antiretroviral drugs in contemporary ART regimens, islatravir is likely to be an effective treatment option.

## 1. Introduction

Antiretroviral therapy (ART), typically administered as a combination of three or more antiretroviral drugs (ARV) from at least two different drug classes, provides an effective therapeutic tool to control disease progression in people living with HIV (PLWH) [1,2,3]. However, their efficacy is jeopardized by ARV resistant viral variants that emerge during sub-therapeutic ARV exposures, leading to the development of acquired drug resistance [4]. Importantly, some mutations in these resistant viral variants could confer resistance to other ARVs within the same class [5]. This ability of viral variants to development cross-resistance significantly limits ARV options available for ART. The development of novel ARVs with unique inhibitory mechanisms, potentially unaffected by ARV resistant viral variants, remain at the forefront in drug development [6,7].

Islatravir (ISL, MK-8591) is a potent nucleoside reverse transcriptase translocation inhibitor (NRTtI), with a half-life that exceeds that of currently approved nucleoside reverse transcriptase inhibitors (NRTIs) [8,9]. Despite lymphopenia observed at high ISL doses during initial clinical trials [10], subsequent clinical trials using lower ISL doses demonstrated no significant reductions in lymphocyte counts, and ISL was well tolerated with an efficacy that is non-inferior to currently approved ART [11,12]. ISL, co-formulated with doravirine (DOR) as Idvynso™ (Merck & Co., Inc., Rahway, NJ, USA), has recently been approved for the treatment of virologically suppressed PLWH who has no history of virological treatment failure and no known mutations associated with DOR resistance [13]. Due to its longer half-life, ISL in currently under investigation for once-weekly oral dosing, in combination with Lenacapavir [14].

Despite its novel inhibitory mechanism [15], several ISL-associated resistance mutations have been reported and involve mainly M184IV and thymidine analogue mutations (TAMs) [16]. The M184VI mutations are typically selected for by lamivudine (3TC) and emtricitabine (FTC) [17] and M184VI confers high levels of resistance to these ARVs. TAMs are typically selected for by the zidovudine (AZT) and stavudine (d4T) nucleoside analogues. Two distinct TAM resistance pathways have been identified, namely the TAM1 (M41L, L210W, T215Y) and TAM2 (D67N, K70R, T215F, K219EQ) pathways [18]. TAMs confer resistance to AZT and d4T, as well as cross-resistance to abacavir (ABC), didanosine (ddI), and tenofovir (TNF) [5]. The level of resistance depends on the number of accumulated TAM mutations in the viral variant [19].

We previously reported on the impact of NRTI resistance mutations identified in ART experienced PLWH failing ART in South Africa on the in vitro phenotypic susceptibility to ISL [20]. We observed high levels of ISL resistance for some combinations of M184V with type-2 TAMs (TAM2), while M184V in isolation inferred a lower level of ISL resistance. Since all PLWH in the cohort failed lamivudine 3TC and/or emtricitabine FTC-containing regimens at some point, which selects for the M184V mutation, no TAM2 combinations were observed in the absence of M184V. The magnitude of the contribution of M184V to ISL resistance when in combination with TAM2 mutations is therefore unclear. Moreover, it has been shown that the M184V mutation could augment ISL resistance when in combination with other mutations (e.g., A114S [21]); therefore, the current study investigated whether this also applies to TAM2 combinations.

## 2. Materials and Methods

### 2.1. Vectors Used in This Study

The HIV-1 wildtype Gag-Pol expression vector (i.e., p8.9MJ4) was kindly provided by Didier Trono (École Polytechnique Fédérale de Lausanne, Lausanne, Switzerland) and Deenan Pillay (University College London, London, UK). In addition, three HIV-1 Gag-Pol expression constructs (i.e., pRT72, pRT73, and pRT106) were available in our laboratory and were used as M184V + TAM2 control constructs for this study. These constructs were previously generated and subsequently identified as producing HIV-1 subtype C pseudoviruses that exhibited the highest levels of in vitro resistance to ISL among the panel of pseudoviruses that were tested [20]. Specifically, pRT72 contained D67N, K70R, M184V, and K219E; pRT73 contained D67N, K70R, M184V, T215F, and K219E; pRT106 contained D67N, K70R, M184V, and K219Q. All three constructs were previously generated using PCR-based site-directed mutagenesis, with the wildtype p8.9MJ4 serving as the parental backbone [20]. The M184V construct consisted of the p8.9MJ4 wildtype backbone that was previously modified to contain only the M184V mutation in the absence of TAM2s.

The pCSFLW firefly luciferase transfer vector was kindly provided Nigel Temperton (Medway School of Pharmacy, Gillingham, United Kingdom). The pHEF Vesicular Stomatitis Virus glycoprotein (VSV-G) expressing vector was obtained from through the NIH HIV Reagent Program, Division of AIDS, NIAID, NIH (ARP-4693, contributed by Dr. Lung-Ji Chang).

ISL was purchased from MedChemExpress (Ipswich, MA, USA; Cat. No. HY-104012) and prepared in dimethyl sulfoxide (DMSO) at 10 mM. Aliquots of the stock were stored at −80 °C. Working solutions of 1 mM were prepared by dilution in complete DMEM.

### 2.2. Modification of HIV-1 Gag-Pol Expression Vectors

The three HIV-1 Gag-Pol expression constructs were used as the backbone vectors to revert the M184V mutation to wildtype (i.e., 184M) through PCR-based site-directed mutagenesis using the following forward and reverse primers, respectively: M184V_Rev_F (5′-CAG AAA TAG TCA TCT ATC AAT ATA TGG ATG ATT TGT ATG TAG G-3′) and M184V_Rev_R (5′-CCT ACA TAC AAA TCA TCC ATA TAT TGA TAG ATG ACT ATT TCT GG-3′). For the site-directed mutagenesis reactions, an approach similar to that published by Yang et al. (2022) [22] was followed, where overlapping primers for the ampicillin resistance gene were used: Amp-F (5′-CAG CGT TTC TGG GTG AGC AAA AAC AG-3′) and Amp-R (5′-CTGTTTTTGCTCACCCAGAAACGC-3′). In this approach, the plasmid was amplified in two separate PCR reactions, with each reaction containing a combination of an M184V_Rev primer and an Amp primer (M184V_Rev_F + Amp-R or M184V_Rev_R + Amp-F). The two resulting PCR amplicons contain overlapping ends that can be joined through homologous recombination after transformation of competent bacterial cells.

The PCR reactions were performed by combining 12.5 μL of NEB Q5 Mastermix (2×, New England Biosciences, Ipswich, MA, USA), 1.25 μL forward primer (10 μM), 1.25 μL reverse primer (10 μM), 1 μL plasmid DNA (13 ng/μL; 12,151 bp), and 9.0 μL water to a final volume of 25 μL. Thermocycling was performed as follows: Initial denaturation at 98 °C for 30 s, followed by 30 cycles of denaturation at 98 °C for 10 s, annealing at 62 °C for 30 s, and elongation at 72 °C for 6 min. A final elongation step was performed at 72 °C for 2 min, followed by a holding step at 15 °C. PCR reactions were analyzed through electrophoresis on a 1% agarose gel to confirm amplicon sizes (i.e., 5517 bp and 6634 bp, respectively).

Chemically competent DH5α bacterial cells (50 μL) were transformed with 2.5 μL from each the two matching PCR reactions through standard heat-shock. After the addition of 450 μL super optimal broth (SOB), the bacteria were incubated at 37 °C with shaking at 220 rpm for 1 h. The bacteria were then pelleted by centrifugation at 4000 rpm for 1 min, after which 400 μL of supernatant was removed and discarded. The bacterial pellets were resuspended in the remaining 50 μL of SOB and plated on ampicillin (100 μg/mL)-containing agar plates. The agar plates were incubated at 37 °C overnight, after which colonies were picked and inoculated into 4 mL of Luria–Bertani (LB) broth containing ampicillin (100 μg/mL). Bacterial cultures were incubated at 37 °C with shaking at 220 rpm for approximately 16 h. Bacterial cells were pellet through centrifugation at 3000 rpm for 10 min, after which the supernatant was discarded, and plasmid DNA was extracted from bacterial pellets using the Zyppy™ Plasmid Miniprep Kit (Zymo Research, Irvine, CA, USA). Plasmid DNA was quantified on a NanoDrop One (ThermoFisher Scientific, Waltham, MA, USA). The plasmid DNA was submitted to Sanger sequencing using the BigDye™ Terminator v3.1 Cycle Sequencing Kit (ThermoFisher Scientific, Waltham, MA, USA), and DNA analysis was performed on an ABI Genetic Analyzer (ThermoFisher Scientific, Waltham, MA, USA). Sequence read analysis was performed using Geneious Prime version 10.2.6 (Dotmatics, Boston, MA, USA). Contiguous sequences were submitted to the Stanford HIVdb Program (https://hivdb.stanford.edu/; accessed on 1 September 2025) [23] to confirm the presence of TAM2s and absence of M184V.

### 2.3. Preparation and Titration of HIV-1-like Pseudoviruses

Pseudoviruses from all eight HIV-1 Gag-Pol constructs were prepared through co-transfection of HEK293T cells. The cells were maintained in high-glucose (4.5 g/L) Dulbecco’s Modified Eagles Medium (DMEM; Capricorn Scientific, Ebsdorfergrund, Germany) with glutamine (0.6 g/L), supplemented with 10% fetal bovine serum (Merck, Darmstadt, Germany), sodium pyruvate (0.11 g/L), HEPES (25 mM), and gentamycin (50 μg/mL). On the day prior to transfection, 8 × 10^6^ HEK293T cells were seeded in 10 mL complete DMEM in a 10 cm Nunc™ Nuclon Delta culture dishes (ThermoFisher Scientific, Waltham, MA, USA) and incubated at 37 °C under 5% CO_2_ in a humidified atmosphere. For the transfection, the following vectors were combined: 1.5 μg HIV-1 gag-pol expression vector, 1.0 μg pCSFLW, and 0.25 μg pHEF-VSV in 50 μL Opti-MEM™ reduced serum medium (ThermoFisher Scientific, Waltham, MA, USA). Afterwards, 8.25 μL PEImax (1 mg/mL; Polysciences^®^, Warrington, PA, USA) was added to each transfection mix and incubate for 20 min at room temperature. After replacement of the medium in the culture dishes with fresh complete DMEM, the transfection mixes were added dropwise and the dishes gently swirled to distribute the DNA complexes. The culture dishes were incubated at 37 °C under 5% CO_2_ in a humidified atmosphere for 48 h, after which the supernatants were collected in 15 mL conical tubes. The tubes was centrifuged at 2000 rpm for 10 min to pellet cells and cell debris, after which the supernatants were divided into 1 mL aliquots and stored at −80 °C.

The resulting HIV-1-like pseudoviruses contain a firefly luciferase RNA transcript encased in the HIV-1 capsid, together with the viral enzymes (Reverse Transcriptase, Integrase, Protease). Vesicular Stomatitis Virus Glycoproteins (VSV-G) are displayed on the pseudoviral membranes to facilitate infection and entry into the target cell. Post-infection, the luciferase RNA transcript is transcribed by the HIV-1 Reverse Transcriptase (RT) and integrated into the host’s genome by HIV-1 Integrase. The integrated luciferase gene is expressed under control of the spleen focus-forming virus promoter to produce luciferase that can be assayed through the addition of luciferin substrate. The inhibition of RT by ISL results in a decrease in the expression of luciferase.

The pseudoviruses were titered in HEK293T cells by preparing eight two-fold serial dilutions of harvested stocks in a final volume of 50 μL complete DMEM in Nunc™ Edge 2.0 96-well culture plates (ThermoFisher Scientific, Waltham, MA, USA). Fifty microliters of HEK293T cells (4 × 10^5^ cells/mL) were added to the wells, and the plates were incubated at 37 °C under 5% CO_2_ in a humidified atmosphere for 72 h. Luciferase expression was assessed using the Bright-Glo™ Luciferase Assay System (Promega, Madison, WI, USA) and bioluminescence was quantified on a GloMax^®^ Explorer microplate reader (Promega, Madison, WI, USA). Pseudoviral inputs for the in vitro phenotypic drug susceptibility assays were standardized at dilutions that provided a bioluminescence of 1 × 10^6^ relative light units (RLU) in the no-drug control.

### 2.4. In Vitro Phenotypic Drug Susceptibility Testing

To assess the level of ISL resistance of the different pseudovirus mutants, eleven three-fold serial dilutions of ISL in duplicate was prepared in duplicate in 50 μL complete DMEM per well in Nunc™ Edge 2.0 96-well culture plates. HEK293T cells were combined with the appropriate dilution of pseudovirus in complete DMEM at 4 × 10^5^ cells/mL, after which 50 μL was added to the wells containing the ISL dilutions. The final ISL concentrations ranged from 5.0 × 10^−1^ to 8.5 × 10^−6^ μM. Wells containing no ISL (no-drug control) were included as control for full viral activity (i.e., no inhibition). Each assay included a wildtype pseudovirus (i.e., no drug resistance mutations), in addition to the mutant pseudoviruses (i.e., M184V, RT72, RT72_Rev, RT73, RT73_Rev, RT106, RT106_Rev). The plates were incubated at 37 °C under 5% CO_2_ in a humidified atmosphere for 72 h, after which the expression of luciferase was assessed with the Bright-Glo™ Luciferase Assay System and GloMax^®^ Explorer microplate reader.

For each pseudovirus, viral activity was calculated at each ISL dilution by division of the RLU value by that of the no-drug control to yield percent viral activity. The inhibitory concentration-50s (IC_50_) were calculated in Microsoft Excel (Microsoft, Redmond, WA, USA) using the FORECAST formula. The fold change (FC) difference in IC_50_ between the mutant and wildtype pseudoviruses were calculated by dividing the IC_50_ of the mutant pseudovirus by the IC_50_ of the wildtype pseudovirus. Each pseudovirus was screened in ≥9 independent phenotypic assays.

### 2.5. Statistical Analysis

Comparison of the FC values between the different pseudoviruses was performed in R Studio (version 2026.07.1, Build 147) [24] using the tidyverse [25], rstatix [26] and PMCMRplus [27] packages. The data was assessed for normality in distribution and equality in variance before the appropriate multiple comparisons test was performed, as suggested by Gurski and Dittel [28]. Differences in FC values were deemed statistically significant if the *p*-values were <0.05. The figure was produced in R Studio using the ggplot2 package [29], with error bars indicating standard error of the means (SEM).

## 3. Results

**Figure 1:**
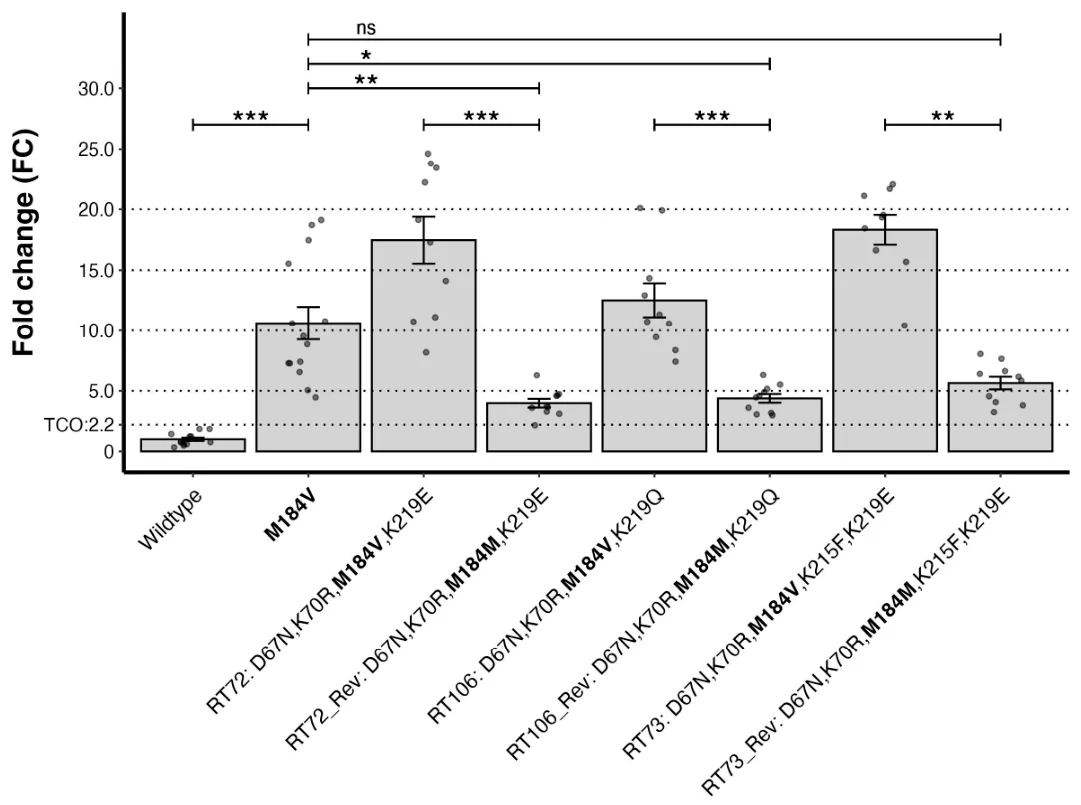
ISL sensitization of TAM2 combinations after reversion of M184V. HIV-1 subtype C Gag-Pol expression vectors, which contained various TAM2 mutations in combination with the M184V mutation, were subject to PCR-based site-directed mutagenesis to revert the M184V mutation back to the wildtype (i.e., M184). The expression vectors were subsequently used to generate pseudoviruses through transfection of HEK293T cells. Pseudoviruses were then assessed for ISL susceptibility in an in vitro single-cycle luciferase-based reporter assay. Their respective IC_50_ values were calculated and expressed as fold-change (FC) difference in IC_50_ to the wildtype reference. The lower technical cutoff (TCO) for ISL (i.e., FC 2.2) has previously been as established for this assay [16] and is indicated on the y-axis in the figure. The Shapiro–Wilk test indicated that FC values for were normally distributed (*p* = 0.0062 to 0.735) for all variants. A Brown–Forsythe test showed a significant inequality (*p* = 0.00000491) in variance between the FC values of the variants. Subsequently, a Welch’s ANOVA showed evidence of a significant difference (*p* = 2.94 × 10^−16^) in FC values between the variants. A pairwise multiple comparisons of mean rank sums extended test (i.e., Dunnett T3 test) was then performed to assess significance of differences in the FC values between the variants. The statistical significances in the differences are indicated as horizontal bars on the figure: ns (not significant), * (*p* < 0.050), ** (*p* < 0.010), *** (*p* < 0.001). Error bars represent standard errors of the means (SEM).

The pseudovirus expressing the single M184V mutation exhibited a significant decrease in ISL susceptibility, with an average fold-change of 10.6 (±5.1 SD, *p* = 0.0002) relative to the wildtype pseudovirus lacking drug resistance-associated mutations (Figure 1, Tables S1 and S2). This finding established M184V as a major determinant of reduced ISL susceptibility, independent of TAM2 background.

All three pseudoviruses expressing TAM2 mutations in combination with M184V (RT72, RT106, and RT73) showed significant further reductions in ISL susceptibility beyond that observed with M184V: RT72 (FC 17.4 ± 6.0 SD, *p* = 0.0003), RT106 (FC 12.5 ± 4.4 SD, *p* = 0.0004), and RT73 (FC 18.3 ± 3.6 SD, *p* = 7.5 × 10^−6^). Notable, no statistically significant differences in ISL susceptibility were observed among these three TAM2 + M184V-containing pseudoviruses (*p* > 0.131; Table S2).

Reversion of mutant 184V to wildtype M184 in each of the three TAM2-containing constructs resulted in marked and statistically significant increases in ISL susceptibility. Compared to their respective M184V-containing variants, the fold-change values of the M184V-revertants were 13.4-fold lower for RT72_Rev (FC 3.9 ± 1.1 SD, *p* = 0.0010), 12.7-fold lower for RT106_Rev (FC 4.3 ± 1.1 SD, *p* = 0.0048), and 8.1-fold lower for RT73_Rev (FC 5.6 ± 1.6 SD, *p* = 1.9 × 10^−6^), demonstrating that M184V is the primary driver of resistance in TAM2 + M184V genotypes. Both the RT72_Rev and RT106_Rev pseudoviruses, which lacked M184V, were significantly more susceptible to ISL than the single M184V pseudovirus (*p* = 0.0063 and *p* = 0.0110, respectively). Although RT73_Rev showed a similar trend towards increased susceptibility relative to M184V alone, this difference approached but did not achieve statistical significance (*p* = 0.0705).

Despite the significant increase in ISL susceptibility following the reversion of 184V to M184, all three TAM2_Rev pseudoviruses remained significantly (*p* < 0.0002) less susceptible to ISL than the wildtype pseudovirus without drug resistance mutations (*p* < 0.0003). This observation indicates that, while M184V is the primary determinant of ISL resistance in TAM2 + M184V genotypes, TAM2s retain measurable independent contributions to the ISL-resistant phenotype even in the absence of M184V.

## 4. Discussion

In vitro phenotypic ISL susceptibility of pseudoviruses expressing various combinations of TAM2 and M184V mutations was systematically assessed in a single cycle luciferase-based reporter assay. Our results demonstrate two principal findings: (1) pseudoviruses expressing either M184V alone or in combination with TAM2 mutations showed significant reductions in ISL susceptibility, and (2) reversion of mutant M184V to wildtype M184 produces substantial ISL sensitization, although phenotypic resistance persists in the TAM2 background alone, although still showing significantly reduced ISL susceptibility compared to the wildtype pseudovirus. Thus, M184V is a major determinant of the magnitude of ISL susceptibility, with its effect modified by the surrounding TAM2 genetic background.

Previous studies have elucidated the two broad mechanisms of NRTI resistance [30], which is typically facilitated through either (i) a discriminatory mechanism (e.g., mediated through K65R and/or M184V) in which RT sterically excludes an NRTI from the active site while still allowing the analogous natural dNTP substrate to enter the active site, or (ii) an excisionary mechanism (e.g., mediated through TAMs) through the phosphorolytic removal of an incorporated chain-terminating NRTI residue from the 3′-end of the growing viral DNA strand. These two resistance mechanisms are considered to be mutually exclusive [31] and can lead to antagonism when the related mutations occur together, as exemplified by the well-characterized bidirectional antagonism between K65R and TAMs [32]. In addition, prior studies have demonstrated that M184V reduces both the accumulation of TAMs and the phenotypic resistance to thymidine analogues (i.e., zidovudine and stavudine) [33,34]. The observations presented in the current study directly contradict this established paradigm by providing robust phenotypic evidence consistent with cooperation, instead of antagonistic interactions, between M184V and TAM2 mutations in the context of ISL resistance. To our knowledge, no prior studies have investigated the mechanistic interactions between M184V and TAMs in this context, representing a notable gap in our understanding of ISL resistance development.

ISL possesses a mechanistically distinct action profile amongst NRTIs, inhibiting reverse transcription through three distinct mechanisms [15]: (i) immediate chain termination, which prevents the addition and incorporation of deoxynucleoside triphosphates (dNTPs) into the growing DNA strand; (ii) delayed chain termination, which allows the incorporation of one additional nucleotide before chain termination occurs; and (iii) misincorporation, which interrupts primer extension and critically, precludes removal of the incorporated drug via the excisionary mechanism [35]. Importantly, the last two features represent key distinctions from conventional NRTIs.

Based on our phenotypic findings, the established mechanisms of NRTI resistance, and the different modes of ISL inhibition, we hypothesize that M184V and TAM2s may reduce ISL susceptibility through complementary actions, rather than competing mechanisms. Specifically, M184V likely diminishes ISL incorporation through nucleotide discrimination, the same mechanism by which it confers resistance to 3TC and FTC [36], thereby reducing the pool of incorporated ISL. Concurrently, TAM2 mutations may promote the removal of incorporated ISL following immediate chain termination and, more likely, during enzyme pausing associated with delayed chain termination.

Importantly, the misincorporation of ISL could partially counteract TAM2-mediated excision, since misincorporated ISL cannot be readily removed by the phosphorolytic activity of TAM-containing RT [35]. This complex interplay between M184V-mediated discrimination, TAM2-mediated excision, and ISL-specific misincorporation may also account for the some of the variability observed in our single-cycle phenotypic assay, particularly among M184V-containing pseudoviruses. Within this framework, variation between experimental replicates may reflect stochastic differences in the relative contributions of each of the resistance mechanisms to the overall phenotype.

Although the mechanistic explanation presented above is plausible and consistent with our phenotypic findings, the hypothesis remains fundamentally speculative and requires direct biochemical validation to determine the quantitative contribution of each putative mechanism to the observed phenotype. Appropriate biochemical assays, such as those as employed by Michailidis et al. (2014) [35], would need to be performed on TAM2-containing RT enzymes with and without M184V to validate this hypothesis. These assays would shed light on the contribution of TAM2s and M184V in resistance to ISL at the immediate and late chain termination steps, as well as ISL misincorporation and excision.

Elucidating the molecular basis of M184V + TAM2 cooperation in ISL resistance has substantial implications for clinical practice. A comprehensive mechanistic understanding would not only clarify the molecular underpinnings of ISL resistance but would also enable more accurate prediction of resistance in treatment-experienced PLWH and substantially improve the interpretation of complex genotypic resistance profiles as ISL-containing antiretroviral regimens are introduced into clinical practice. These insights will be essential for optimal patient stratification and therapeutic decision-making in future ISL-based treatment strategies.

In light of the continued evolution of ART regimens, especially in low-to-middle income countries, however, ISL is expected to remain a potentially effective treatment option for PLWH either initiating or failing regimens that do not included the TAM-selecting ARVs (i.e., AZT, d4T). In South Africa, d4T was replaced with TDF in adult treatment regimens and by ABC in pediatric regimens in 2010 [37]. More recently, AZT has been removed from standard ART regimens and is reserved for specific clinical scenarios [38], while the effective recycling of TDF and 3TC/FTC in DTG-based second-line therapy [39,40] will further reduced reliance on TAM-selecting ARVs (i.e., AZT, d4T). Consequently, the continued reduction in the use of AZT and d4T will limit the future emergence of TAMs, supporting the potential use of ISL in PLWH failing contemporary ART regimens. However, ISL may be less effective in treatment-experienced PLWH previously exposed to historic AZT- and/or d4T-containing regimens, in whom archived viruses harboring TAM2 mutations together with M184V may persist.

## 5. Conclusions

Our findings demonstrate that M184V can substantially enhance the impact of TAM2s on ISL resistance, highlighting the importance of considering pre-existing NRTI resistance when including ISL in treatment regimens for treatment experienced PLWH, particularly those with extensive NRTI resistance failing historic ART regimens.

## Data Availability

The original contributions presented in this study are included in the article/Supplementary Materials; further inquiries can be directed to the corresponding author.

## Supplementary Materials

The following supporting information can be downloaded at: https://www.mdpi.com/article/10.3390/v18091046/s1, Table S1: Descriptive statistics of fold-change (FC) values of pseudoviruses; Table S2: Dunnett’s T3 test results for multiple comparison of FC values.

## Abbreviations

The following abbreviations are used in this manuscript:

3TC	Lamivudine
ABC	Abacavir
ANOVA	Analysis of variance
ART	Antiretroviral therapy
ARV	Antiretroviral drug
AZT	Zidovudine
d4T	Lamivudine
ddI	Didanosine
DMEM	Dulbecco’s modified Eagle medium
DOR	Doravirine
dNTP	Deoxyribonucleotide triphosphate
FC	Fold change
FTC	Emtricitabine
HIV-1	Human immunodeficiency virus type-1
HEK	Human embryonic kidney
HEPES	4-(2-hydroxyethyl)-1-piperazineethanesulfonic acid
IC_50_	Inhibitory concentration-50
ISL	Islatravir
_Rev	Reversion of M184V to M184)
LB	Luria-Bertani
NNRTI	Non-nucleoside reverse transcriptase inhibitor
NRTI	Nucleos(t)ide reverse transcriptase inhibitor
NRTtI	Nucleoside reverse transcriptase translocation inhibitor
PCR	Polymerase chain reaction
PEImax	Polyethylenimine-max
PLWH	People living with HIV
RLU	Relative light units
RT	Reverse transcriptase
SD	Standard deviation
SEM	Standard error of the mean
SOB	Super optimal broth
TAM2	Thymidine analogue mutation type 2
TCO	Technical cutoff
TNF	Tenofovir
VSV-G	Vesicular stomatitis virus glycoprotein
