# Supplementary material for: M184V: The Major Determinant of Cooperative ISL Resistance When in Combination with Multiple TAM2 Mutations in HIV-1 Subtype C
Source: Viruses. 2026 Sep 20;18(9):1046. doi: 10.3390/v18091046 (PMC13611880; doi:10.3390/v18091046)
Supplement: Supplementary file 1 [file viruses-18-01046-s001.zip › viruses-4551845-supplementary.pdf]

Supplementary:

Supplementary tables

Table S1: Descriptive statistics of fold-change (FC) values of pseudoviruses

*Variant:	Wildtype	M184V	RT72	RT72_KO	RT73	RT73_KO	RT106	RT106_KO
**Average**	1.00	10.62	17.46	3.98	18.32	5.64	12.52	4.37
**SD**	0.49	5.05	6.10	1.13	3.67	1.65	4.43	1.14
**SEM**	0.14	1.35	1.93	0.36	1.22	0.52	1.40	0.36
**Min**	0.33	4.46	8.18	2.15	10.42	3.24	7.41	2.98
**1stQ**	0.64	7.27	11.87	3.36	16.64	4.19	9.74	3.28
**Mean**	0.82	9.20	18.21	3.72	19.33	6.00	11.05	4.52
**3rdQ**	1.23	14.35	23.12	4.61	21.10	6.57	13.98	5.09
**Max**	1.85	19.12	24.62	6.28	22.05	8.05	20.10	6.31
**IQR**	0.60	7.07	11.25	1.26	4.46	2.39	4.24	1.81

*RT72: D67N, K70R, M184V, K219E; RT72_KO: D67N, K70R, K219E; RT73: D67N, K70R, M184V, T215F, K219E; RT73_KO: D67N, K70R, T215F, K219E; RT106: D67N, K70R, M184V, K219Q; RT106_KO: D67N, K70R, K219Q. "_KO" signifies reversion of the mutant 184V to the wildtype 184M.

Table S2: Dunnett's T3 test results for multiple comparison of FC values

*Variant	WT	M184V	RT72	RT72_KO	RT73	RT73_KO	RT106
**M184V**	0.00019	-	-	-	-	-	-
**RT72**	0.00028	0.19397	-	-	-	-	-
**RT72_KO**	0.00010	0.00635	0.00100	-	-	-	-
**RT73**	1.2e-05	0.00974	1.00000	5.5e-05	-	-	-
**RT73_KO**	0.00014	0.07045	0.00329	0.32058	3.7e-05	-	-
**RT106**	0.00040	0.99979	0.66031	0.00333	0.13156	0.01660	-
**RT106_KO**	6.1e-05	0.01095	0.00128	0.99999	2.8e-05	0.71372	0.00480

*RT72: D67N, K70R, M184V, K219E; RT72_KO: D67N, K70R, K219E; RT73: D67N, K70R, M184V, T215F, K219E; RT73_KO: D67N, K70R, T215F, K219E; RT106: D67N, K70R, M184V, K219Q; RT106_KO: D67N, K70R, K219Q. "_KO" signifies reversion of the mutant 184V to the wildtype 184M.
